# Supplementary material for: Longitudinal associations between time perspective and life satisfaction across adulthood
Source: Commun Psychol. 2024 Jul 20;2:67. doi: 10.1038/s44271-024-00118-0 (PMC11332047; doi:10.1038/s44271-024-00118-0)
Supplement: Supplementary file 2 — Supplementary Information [file 44271_2024_118_MOESM2_ESM.pdf]

Supplementary Table 1

Demographic information of AAF participants by cohort

Demographics	1929–1938 cohort (n = 78)	1939–1948 cohort (n = 98)	1949–1958 cohort (n = 102)	1959–1968 cohort (n = 100)	1969–1978 cohort (n = 81)
Sex (%)					
female	43 (55.1)	52 (53.1)	54 (52.9)	42 (42.0)	43 (53.1)
male	35 (44.9)	46 (46.9)	48 (47.1)	58 (58.0)	38 (46.9)
Primary education (%)					
< 10 years	24 (30.8)	25 (25.5)	9 (8.8)	8 (8.0)	3 (3.7)
10 years	44 (56.4)	47 (48.0)	55 (53.9)	52 (52.0)	65 (80.2)
> 10 years	9 (11.5)	26 (26.5)	37 (36.3)	37 (37.0)	13 (16.1)
missing	1 (1.3)	0 (0)	1 (1.0)	3 (3.0)	0 (0)
Secondary Education (%)					
vocational training	14 (18.0)	20 (20.4)	25 (24.5)	30 (30.0)	17 (21.0)
vocational college	25 (32.1)	29 (29.6)	22 (21.6)	21 (21.0)	16 (19.7)
College	12 (15.4)	14 (14.3)	19 (18.6)	10 (10.0)	17 (21.0)
University	21 (26.9)	34 (34.7)	34 (33.3)	33 (33.0)	28 (34.6)
other	3 (3.8)	1 (1.0)	1 (1.0)	3 (3.0)	3 (3.7)
missing	3 (3.8)	0 (0)	1 (1.0)	3 (3.0)	0 (0)
Subjective health (*SD*)[a]	2.49 (0.92)	2.68 (0.75)	2.59 (1.05)	2.88 (0.91)	3.05 (0.83)
Household income (*SD*)[b]	2146 (893)	2464 (1238)	3035 (1511)	3218 (1994)	2849 (1226)

Note. [a] Subjective health was assessed by a single item, “How would you rate your current health?” with a response scale from 0 (very poor) to 4 (very good). [b] Household income was assessed in Euro, individuals with an income that was more than 3 SD above the mean income were excluded from the calculation of mean income for each age group (*N* = 2).

Supplementary Note 1

Model equations with (1) age vs. (2) SRLE as moderators

$$\text{Life satisfaction}_{it} = \gamma_{00} + \gamma_{01}(\text{wpconc}_{it}) + \gamma_{02}(\text{bpconc}_{i}) + \gamma_{03}(\mathit{wpobs}_{it}) + \gamma_{04}(\mathit{bpobs}_{i}) + \gamma_{05}(\mathit{wppast}_{it}) + \gamma_{06}(\mathit{bppast}_{i}) + \gamma_{07}(\mathit{wpfinit}_{it}) + \gamma_{08}(\mathit{bpfinit}_{i}) + \gamma_{09}(\text{wpconc}_{it} * \text{age}_{i}) + \gamma_{10}(\text{bpconc}_{i} * \text{age}_{i}) + \gamma_{11}(\text{wpobs}_{it} * \text{age}_{i}) + \gamma_{12}(\text{bpobs}_{i} * \text{age}_{i}) + \gamma_{13}(\text{wppast}_{it} * \text{age}_{i}) + \gamma_{14}(\text{bppast}_{i} * \text{age}_{i}) + \gamma_{15}(\text{wpfinit}_{it} * \text{age}_{i}) + \gamma_{16}(\text{bpfinit}_{i} * \text{age}_{i}) + \gamma_{17}(\text{age}_{i}) + \gamma_{18}(\text{sex}_{i}) + \gamma_{19}(\text{educationdummy1}_{i}) + \gamma_{20}(\text{educationdummy2}_{i}) + \gamma_{21}(\text{educationdummy3}_{i}) + \gamma_{22}(\text{income}_{i}) + \gamma_{23}(\text{health}_{i}) + \gamma_{24}(\text{time}_{it}) + u_{0i} + u_{1i}\text{time}_{it} + r_{it} \quad (1)$$

$$\text{Life satisfaction}_{it} = \gamma_{00} + \gamma_{01}(\text{wpconc}_{it}) + \gamma_{02}(\text{bpconc}_{i}) + \gamma_{03}(\mathit{wpobs}_{it}) + \gamma_{04}(\mathit{bpobs}_{i}) + \gamma_{05}(\mathit{wppast}_{it}) + \gamma_{06}(\mathit{bppast}_{i}) + \gamma_{07}(\mathit{wpfinit}_{it}) + \gamma_{08}(\mathit{bpfinit}_{i}) + \gamma_{09}(\text{wpconc}_{it} * \text{SRLE}_{i}) + \gamma_{10}(\text{bpconc}_{i} * \text{SRLE}_{i}) + \gamma_{11}(\text{wpobs}_{it} * \text{SRLE}_{i}) + \gamma_{12}(\text{bpobs}_{i} * \text{SRLE}_{i}) + \gamma_{13}(\text{wppast}_{it} * \text{SRLE}_{i}) + \gamma_{14}(\text{bppast}_{i} * \text{SRLE}_{i}) + \gamma_{15}(\text{wpfinit}_{it} * \text{SRLE}_{i}) + \gamma_{16}(\text{bpfinit}_{i} * \text{SRLE}_{i}) + \gamma_{17}(\text{SRLE}_{i}) + \gamma_{18}(\text{sex}_{i}) + \gamma_{19}(\text{educationdummy1}_{i}) + \gamma_{20}(\text{educationdummy2}_{i}) + \gamma_{21}(\text{educationdummy3}_{i}) + \gamma_{22}(\text{income}_{i}) + \gamma_{23}(\text{health}_{i}) + \gamma_{24}(\text{time}_{it}) + u_{0i} + u_{1i}\text{time}_{it} + r_{it} \quad (2)$$

Supplementary Table 2

Mean and standard deviations for main study variables by cohort and measurement time point

	1929–1938 cohort			1939–1948 cohort			1949–1958 cohort			1959–1968 cohort			1969–1978 cohort		
	T1	T2	T3	T1	T2	T3	T1	T2	T3	T1	T2	T3	T1	T2	T3
CON	6.69	6.16	6.47	7.70	7.04	7.11	7.89	7.41	7.45	8.70	7.77	8.23	8.52	7.95	8.04
SD	(2.31)	(2.31)	(1.84)	(2.25)	(2.51)	(2.29)	(2.28)	(2.26)	(2.31)	(2.16)	(2.53)	(2.22)	(2.07)	(2.14)	(2.08)
PAST	6.06	5.64	6.62	4.89	5.22	6.04	4.30	4.40	4.48	4.52	4.53	4.46	4.85	4.54	4.81
SD	(2.75)	(2.67)	(2.64)	(2.54)	(2.57)	(2.72)	(2.25)	(2.32)	(2.49)	(2.65)	(2.47)	(2.42)	(2.36)	(2.06)	(2.29)
OBS	3.30	3.75	4.68	2.84	2.99	4.25	2.74	2.92	3.43	2.59	2.95	3.13	3.10	3.44	3.37
SD	(2.22)	(2.56)	(2.52)	(1.97)	(2.05)	(2.29)	(1.98)	(2.04)	(2.47)	(1.92)	(2.17)	(2.23)	(2.31)	(2.15)	(2.32)
FIN	7.91	7.96	8.48	7.42	7.29	7.61	7.26	7.27	7.23	7.21	7.37	7.44	6.64	6.77	6.84
SD	(2.85)	(2.47)	(2.72)	(2.51)	(2.56)	(2.48)	(2.75)	(2.47)	(2.50)	(2.74)	(2.77)	(2.44)	(2.89)	(2.76)	(2.48)
SAT	2.98	2.78	2.71	3.02	2.87	3.01	2.85	2.71	2.86	2.90	2.78	2.91	2.74	2.62	2.77
SD	(0.38)	(0.47)	(0.63)	(0.40)	(0.43)	(0.54)	(0.54)	(0.59)	(0.65)	(0.53)	(0.59)	(0.58)	(0.53)	(0.49)	(0.61)
SWF	3.21	3.09	3.01	3.18	3.02	3.13	3.10	2.97	3.08	3.06	2.93	3.17	2.91	2.58	2.86
SD	(0.57)	(0.72)	(0.76)	(0.58)	(0.65)	(0.64)	(0.71)	(0.69)	(0.81)	(0.80)	(0.89)	(0.69)	(0.84)	(0.96)	(0.80)
SPF	2.83	2.53	2.24	2.97	2.79	2.80	2.63	2.42	2.47	2.79	2.61	2.53	2.52	2.23	2.36
SD	(0.83)	(0.98)	(1.21)	(0.78)	(0.78)	(0.95)	(0.95)	(0.98)	(1.06)	(0.91)	(0.98)	(0.95)	(0.94)	(0.95)	(1.00)
SMF	3.19	3.06	2.96	3.20	2.99	3.16	3.20	2.94	3.06	3.14	2.87	2.98	3.05	2.86	3.04
SD	(0.56)	(0.52)	(0.79)	(0.49)	(0.65)	(0.57)	(0.60)	(0.63)	(0.68)	(0.74)	(0.81)	(0.88)	(0.65)	(0.63)	(0.80)
SHE	2.90	2.50	2.28	2.99	2.76	2.85	2.79	2.54	2.59	2.87	2.74	2.67	2.81	2.67	2.78
SD	(0.80)	(1.11)	(1.15)	(0.73)	(0.87)	(0.93)	(0.96)	(0.97)	(1.12)	(0.87)	(0.94)	(1.00)	(0.85)	(0.97)	(0.97)

Note. SD = standard deviation, CON = concreteness of future time perspective, PAST = orientation towards the past, OBS = feelings of obsolescence, FIN = attitudes toward the finitude of life, SAT = satisfaction with life, SWF = satisfaction with friends, SPF = satisfaction with physical fitness, SCF = satisfaction with mental fitness, SHE = satisfaction with health

Supplementary Figure 1

Model-implied trajectories of the time perspectives

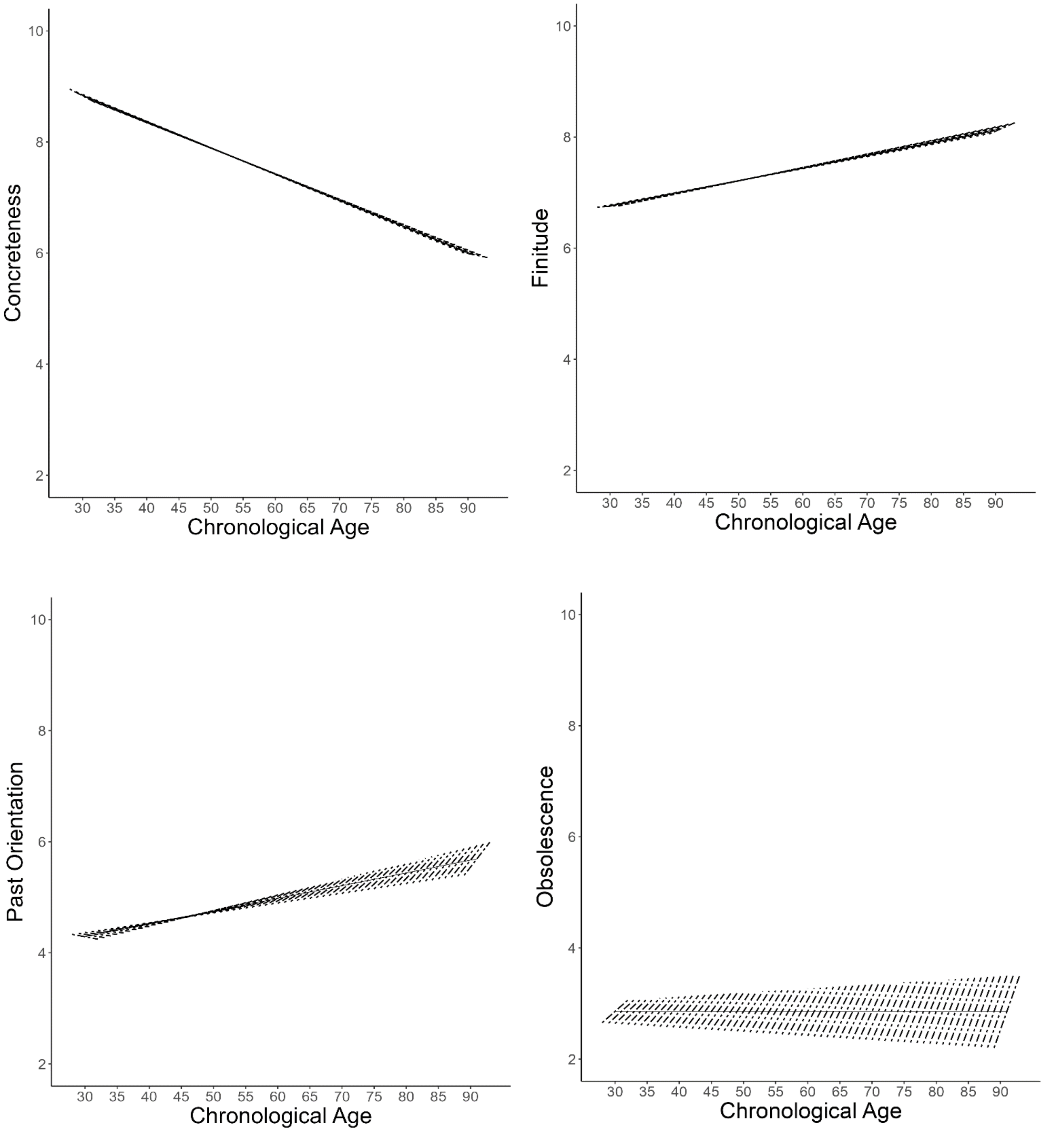

Note. Within-person changes in each time perspective by T1 age are shown as short, thick lines. The cross-sectional linear age trend in each time perspective is shown as a long, thin line. Figures based on syntax used by Gerstorf an d colleagues[43]. n = 459.

Supplementary Figure 2
Between- and within-person associations of concreteness and life satisfaction

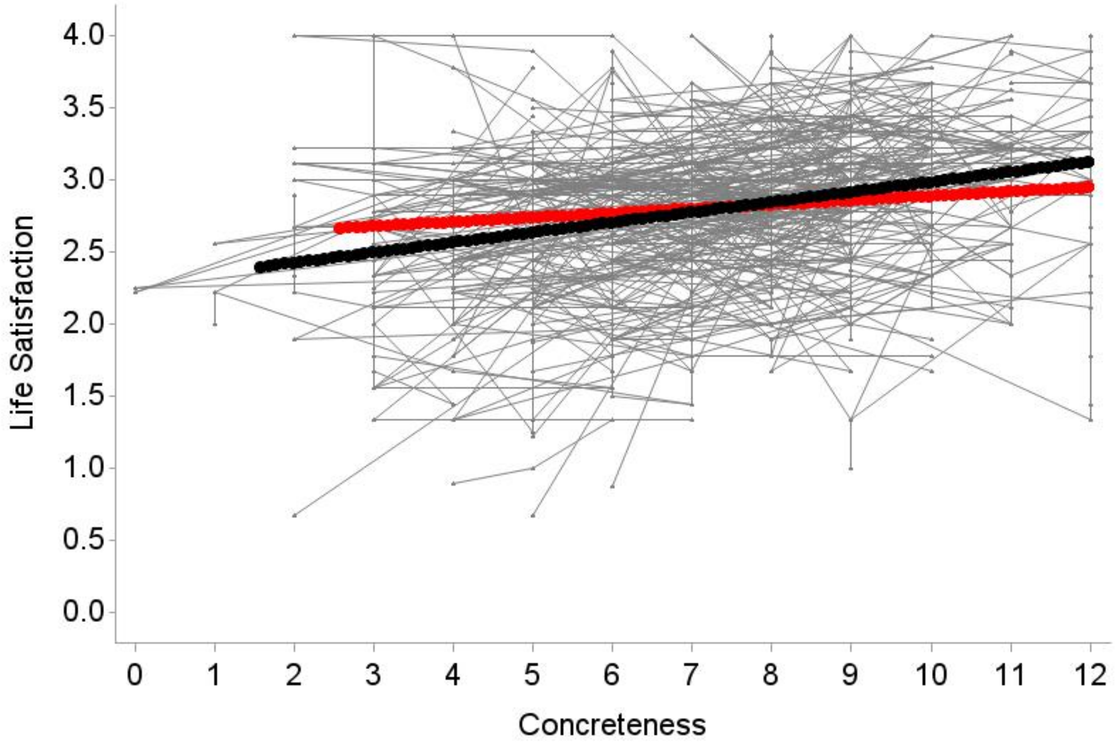

Note. Between-person (black line) and within-person (red line) associations of concreteness and life satisfaction. Grey lines illustrate individual associations. n = 420.

Supplementary Figure 3
Between- and within-person associations of obsolescence and life satisfaction

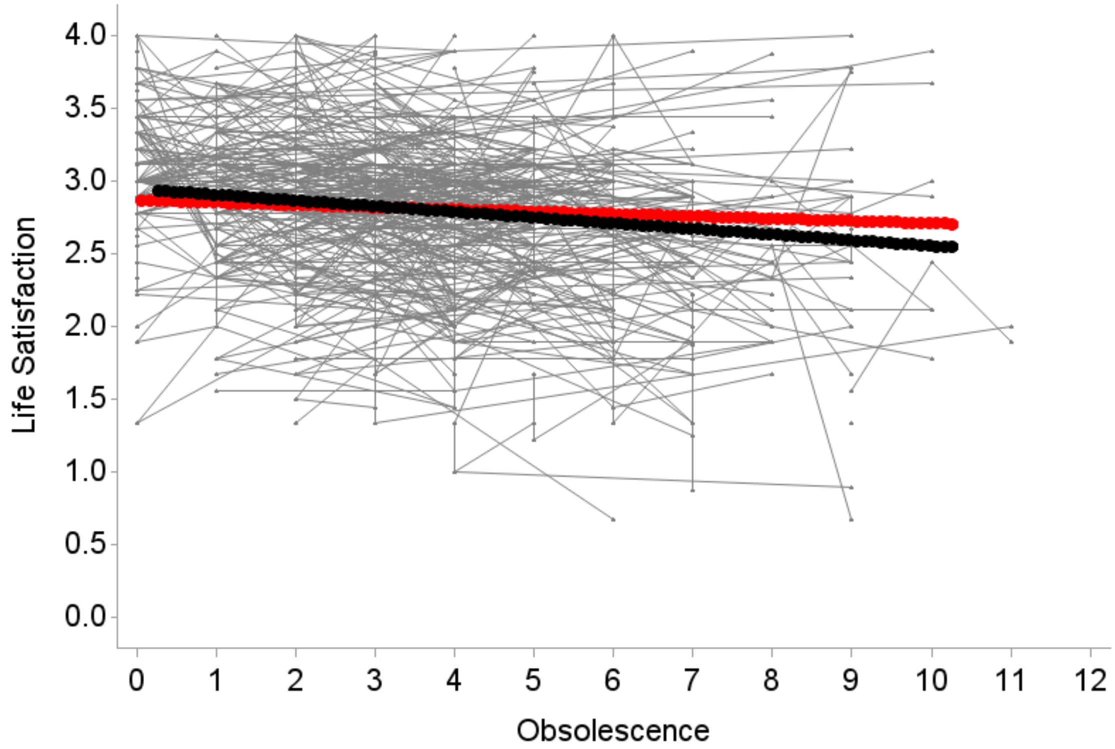

Note. Between-person (black line) and within-person (red line) associations of concreteness and life satisfaction. Grey lines illustrate individual associations. n = 420

Supplementary Table 3

Growth Models of Satisfaction with Friends and Acquaintances, with Time Perspectives as Predictors and (A) Age as Moderator and (B) Subjectively Remaining Life Expectancy (SRLE) as Moderator

	(A)				(B)			
	Estimate	SE	*p*	95% CI	Estimate	SE	*p*	95% CI
Fixed effects								
Intercept	3.030	0.082	<.0001	2.869; 3.192	3.064	0.083	<.0001	2.902; 3.227
Age	0.0108	0.002148	<.0001	0.007; 0.015				
SRLE					-0.009	0.002	<.0001	-0.013; -0.005
Gender	0.121	0.054	0.025	0.015; 0.227	0.117	0.055	0.033	0.010; 0.224
Self-Rated Health	0.072	0.032	0.023	0.010; 0.134	0.075	0.032	0.021	0.011; 0.138
Income	0.015	0.020	0.451	-0.025; 0.055	0.013	0.021	0.529	-0.028; 0.054
Education: Vocational	-0.023	0.086	0.790	-0.193; 0.147	-0.079	0.086	0.357	-0.248; 0.090
College								
Education: College	-0.022	0.104	0.835	-0.226; 0.182	-0.083	0.104	0.424	-0.287; 0.121
Education: University	-0.125	0.084	0.136	-0.289; 0.039	-0.171	0.084	0.042	-0.335; -0.006
bpConcreteness	0.056	0.015	0.0002	0.026; 0.085	0.054	0.015	.0005	0.024; 0.083
bpPast Orientation	0.002	0.015	0.896	-0.027; 0.031	0.005	0.015	0.737	-0.024; 0.035

bpObsolescence	-0.067	0.017	<.0001	-0.101; -0.034	-0.075	0.017	<.0001	-0.109; -0.041
bpFinitude	-0.002	0.013	0.893	-0.025; 0.022	0.001	0.012	0.931	-0.023; 0.025
bpConcreteness*Age	0.002	0.001	0.146	-0.001; 0.004				
bpPast Orientation*Age	0.001	0.001	0.305	-0.001; 0.003				
bpObsolescence*Age	-0.001	0.001	0.549	-0.003; 0.002				
bpFinitude*Age	-0.001	0.001	0.241	-0.003; 0.001				
bpConcreteness*SRLE					-0.000	0.001	0.713	-0.003; 0.002
bpPast Orientation*SRLE					0.000	0.001	0.883	-0.002; 0.002
bpObsolescence*SRLE					-0.000	0.001	0.801	-0.002; 0.002
bpFinitude*SRLE					0.000	0.001	0.583	-0.001;0.002
Time	0.002	0.005	0.650	-0.007; 0.011	0.003	0.004	0.583	-0.007; 0.012
wpConcreteness	0.011	0.013	0.390	-0.014; 0.036	0.012	0.013	0.359	-0.013; 0.037
wpPast Orientation	-0.030	0.01	0.011	-0.054; -0.007	-0.030	0.013	0.014	-0.053; -0.006
wpObsolescence	-0.020	0.013	0.128	-0.046; 0.006	-0.021	0.013	0.111	-0.047; 0.005
wpFinitude	0.018	0.012	0.139	-0.006; 0.043	0.018	0.012	0.154	-0.007; 0.042
wpConcreteness*Age	0.001	0.001	0.346	-0.001; 0.003				
wpPast Orientation*Age	-0.000	0.001	0.938	-0.002; 0.002				

wpObsolescence*Age	0.000	0.001	0.719	-0.001; 0.002				
wpFinitude*Age	-0.001	0.001	0.106	-0.003; 0.000				
wpConcreteness*SRLE					-0.001	0.001	0.111	-0.003;0.000
wpPast Orientation*SRLE					0.001	0.001	0.364	-0.001; 0.002
wpObsolescence*SRLE					-0.000	0.001	0.639	-0.002; 0.001
wpFinitude*SRLE					0.001	0.001	0.134	-0.000; 0.003
Random effects								
Variance intercept	0.169	0.035	<.0001		0.181	0.035	<.0001	
Variance slope	0.000	0.001	.413		0.000	0.001	0.413	
Cov. intercept, slope	-0.002	0.004	.704		-0.002	0.004	0.616	
Variance explained								
	.08				.08			

Note. N = 420 who provided 1,239 observations. bp = between–person. wp = within-person. CI = confidence intervals. Gender was coded as 0 = male, 1 = female. Self-rated health: Higher scores indicate better self-rated health. Education: reference group is vocational training. Income was rescaled in thousands so that a difference in income by one unit corresponds to a difference of 1000 Euro. Cov. = covariance. Unstandardized estimates and standard errors are presented. Chronological age was grand–mean–centered at 54.2 years, and subjective remaining life expectancy was grand-mean centered at 26.6 years.

Supplementary Table 4

Growth Models of Satisfaction with Health, with Time Perspectives as Predictors and (A) Age as Moderator and (B) Subjectively Remaining Life Expectancy (SRLE) as Moderator

	(A)				(B)			
	Estimate	SE	*p*	95% CI	Estimate	SE	*p*	95% CI
Fixed effects								
Intercept	2.851	0.087	<.0001	2.681; 3.022	2.895	0.087	<.0001	2.725; 3.065
Age	0.008	0.002	0.0007	0.003;0.012				
SRLE					-0.004	0.002	0.083	-0.008; 0.000
Gender	0.006	0.057	0.918	-0.107; 0.118	0.006	0.058	0.915	-0.107; 0.120
Self-Rated Health	0.447	0.034	<.0001	0.381; 0.513	0.4445	0.034	<.0001	0.377; 0.512
Income	0.056	0.022	0.010	0.013; 0.099	0.051	0.022	0.020	0.008; 0.094
Education: Vocational	0.002	0.092	0.984	-0.178; 0.182	-0.052	0.091	0.570	-0.230; 0.127
College								
Education: College	-0.126	0.110	0.253	-0.343;0.091	-0.181	0.110	0.101	-0.397: 0.035
Education: University	-0.033	0.089	0.712	-0.207; 0.141	-0.080	0.088	0.365	-0.253; 0.094
bpConcreteness	0.058	0.016	0.0003	0.027; 0.089	0.050	0.016	0.002	0.019; 0.082
bpPast Orientation	-0.005	0.016	0.729	-0.036; 0.025	-0.003	0.016	0.861	-0.034; 0.028

bpObsolescence	-0.021	0.018	0.245	-0.057; 0.015	-0.025	0.018	0.180	-0.060; 0.011
bpFinitude	0.010	0.013	0.413	-0.015; 0.035	0.015	0.013	0.228	-0.010; 0.041
bpConcreteness*Age	-0.000	0.001	0.976	-0.002; 0.002				
bpPast Orientation*Age	0.001	0.001	0.552	-0.002; 0.003				
bpObsolescence*Age	-0.001	0.001	0.311	-0.003; 0.001				
bpFinitude*Age	-0.001	0.001	0.394	-0.002; 0.001				
bpConcreteness*SRLE					0.000	0.001	0.938	-0.002; 0.002
bpPast Orientation*SRLE					-0.001	0.001	0.573	-0.003; 0.002
bpObsolescence*SRLE					0.002	0.001	0.106	-0.000; 0.004
bpFinitude*SRLE					0.001	0.001	0.460	-0.001; 0.002
Time	-0.023	0.006	0.0003	-0.036; -0.011	-0.023	0.006	0.0004	-0.036; -0.011
wpConcreteness	0.020	0.016	0.229	-0.012; 0.051	0.019	0.016	0.246	-0.013; 0.051
wpPast Orientation	-0.023	0.015	0.138	-0.053; 0.007	-0.022	0.015	0.155	-0.052;0.008
wpObsolescence	-0.003	0.017	0.868	-0.036; 0.030	-0.006	0.017	0.703	-0.040; 0.027
wpFinitude	0.005	0.016	0.743	-0.026; 0.036	0.005	0.016	0.760	-0.026; 0.036
wpConcreteness*Age	0.001	0.001	0.290	-0.001; 0.004				
wpPast Orientation*Age	-0.000	0.001	0.995	-0.002; 0.002				

wpObsolescence*Age	-0.000	0.001	0.793	-0.003; 0.002				
wpFinitude*Age	-0.002	0.001	0.131	-0.004; 0.001				
wpConcreteness*SRLE					-0.001	0.001	0.191	-0.004; 0.001
wpPast Orientation*SRLE					0.000	0.001	0.899	-0.002; 0.002
wpObsolescence*SRLE					-0.0001	0.001	0.899	-0.002; 0.002
wpFinitude*SRLE					0.000	0.001	0.743	-0.002; 0.002
Random effects								
Variance intercept	0.059	0.041	.077		0.063	0.042	.065	
Variance slope	0.004	0.001	.003		0.004	0.001	0.003	
Cov. intercept, slope	0.005	0.006	.424		0.005	0.006	0.422	
Variance explained								
	.15				.15			

Note. *N* = 420 who provided 1,239 observations. bp = between–person. wp = within-person. CI = confidence intervals. Gender was coded as 0 = male, 1 = female. Self-rated health: Higher scores indicate better self-rated health. Education: reference group is vocational training. Income was rescaled in thousands so that a difference in income by one unit corresponds to a difference of 1000 Euro. Cov. = covariance. Unstandardized estimates and standard errors are presented. Chronological age was grand–mean–centered at 54.2 years, and subjective remaining life expectancy was grand-mean centered at 26.6 years.

Supplementary Table 5

Growth Models of Satisfaction with Physical Fitness, with Time Perspectives as Predictors and (A) Age as Moderator and (B) Subjectively Remaining Life Expectancy (SRLE) as Moderator

	(A)				(B)			
	Estimate	SE	*p*	95% CI	Estimate	SE	*p*	95% CI
Fixed effects								
Intercept	2.758	0.100	<.0001	2.562: 2.955	2.809	0.101	<.0001	2.611; 3.007
Age	0.015	0.003	<.0001	0.010; 0.021				
SRLE					-0.010	0.003	<.0001	-0.015; -0.005
Gender	-0.042	0.067	0.533	-0.173; 0.089	-0.040	0.068	0.561	-0.173; 0.094
Self-Rated Health	0.362	0.039	<.0001	0.286; 0.439	0.364	0.040	<.0001	0.285; 0.443
Income	0.007	0.025	0.790	-0.043; 0.056	-0.001	0.036	0.978	-0.051;0.050
Education: Vocational	0.025	0.107	0.817	-0.185; 0.235	-0.043	0.107	0.684	-0.253; 0.166
College								
Education: College	-0.023	0.129	0.860	-0.275;0.300	-0.086	0.129	0.507	-0.339; 0.168
Education: University	-0.055	0.103	0.597	-0.258; 0.148	-0.112	0.104	0.282	-0.315; 0.092
bpConcreteness	0.0854	0.019	<.0001	0.049; 0.122	0.076	0.019	<.0001	0.038; 0.113
bpPast Orientation	-0.011	0.018	0.551	-0.047; 0.025	-0.009	0.019	0.619	-0.046; 0.027

bpObsolescence	-0.007	0.021	0.753	-0.048; 0.035	-0.012	0.021	0.575	-0.054; 0.030
bpFinitude	0.003	0.015	0.811	-0.026; 0.033	0.010	0.015	0.491	-0.019; 0.040
bpConcreteness*Age	0.001	0.001	0.668	-0.002; 0.003				
bpPast Orientation*Age	-0.001	0.001	0.525	-0.003; 0.002				
bpObsolescence*Age	0.000	0.001	0.774	-0.002; 0.003				
bpFinitude*Age	-0.002	0.001	0.055	-0.004; 0.000				
bpConcreteness*SRLE					-0.000	0.001	0.908	-0.003; 0.003
bpPast Orientation*SRLE					0.000	0.001	0.731	-0.002; 0.003
bpObsolescence*SRLE					0.001	0.001	0.399	-0.001; 0.004
bpFinitude*SRLE					0.003	0.001	0.015	0.000; 0.005
Time	-0.023	0.006	0.0001	-0.035; -0.011	-0.023	0.006	0.0001	-0.035; -0.012
wpConcreteness	0.022	0.016	0.169	-0.009; 0.052	0.021	0.016	0.176	-0.010; 0.052
wpPast Orientation	-0.029*	0.015	0.046	-0.058; -0.001	-0.029	0.015	0.049	-0.058; -0.000
wpObsolescence	-0.026	0.016	0.118	-0.057; 0.006	-0.028	0.016	0.085	-0.060; 0.004
wpFinitude	0.016	0.015	0.287	-0.014; 0.046	0.0156	0.015	0.302	-0.014; 0.045
wpConcreteness*Age	-0.000	0.001	0.670	-0.003; 0.002				
wpPast Orientation*Age	-0.001	0.001	0.413	-0.003; 0.001				

wpObsolescence*Age	-0.0001	0.001	0.940	-0.002;0.002				
wpFinitude*Age	0.001	0.001	0.305	-0.001; 0.003				
wpConcreteness*SRLE					-0.000	0.001	0.799	-0.002; 0.002
wpPast Orientation*SRLE					0.001	0.001	0.248	-0.001; 0.003
wpObsolescence*SRLE					-0.000	0.001	0.878	-0.002; 0.002
wpFinitude*SRLE					-0.001	0.001	0.160	-0.003; 0.001
Random effects								
Variance intercept	0.149	0.045	0.0005		0.166	0.046	.0002	
Variance slope	0.002	0.001	.081		0.002	0.001	0.077	
Cov. intercept, slope	0.011	0.006	.054		0.011	0.006	0.063	
Variance explained								
	.11				.11			

Note. *N* = 420 who provided 1,239 observations. bp = between–person. wp = within-person. CI = confidence intervals. Gender was coded as 0 = male, 1 = female. Self-rated health: Higher scores indicate better self-rated health. Education: reference group is vocational training. Income was rescaled in thousands so that a difference in income by one unit corresponds to a difference of 1000 Euro. Cov. = covariance. Unstandardized estimates and standard errors are presented. Chronological age was grand–mean–centered at 54.2 years, and subjective remaining life expectancy was grand-mean centered at 26.6 years.

Supplementary Table 6

Growth Models of Satisfaction with Mental Fitness, with Time Perspectives as Predictors and (A) Age as Moderator and (B) Subjectively Remaining Life Expectancy (SRLE) as Moderator

	(A)				(B)			
	Estimate	SE	*p*	95% CI	Estimate	SE	*p*	95% CI
Fixed effects								
Intercept	2.978	0.067	<.0001	2.847; 3.110	3.027	0.068	<.0001	2.893; 3.161
Age	0.009	0.002	<.0001	0.006; 0.013				
SRLE					-0.006	0.002	0.0004	-0.010; 0.003
Gender	0.041	0.044	0.350	-0.045; 0.128	0.044	0.045	0.332	-0.045; 0.133
Self-Rated Health	0.0580	0.026	0.025	0.007; 0.109	0.060	0.027	0.025	0.008; 0.113
Income	-0.004	0.017	0.796	-0.037; 0.028	-0.005	0.017	0.765	-0.039; 0.029
Education: Vocational	0.107	0.070	0.131	-0.032; 0.245	0.04093	0.071	0.565	-0.099; 0.181
College								
Education: College	0.072	0.084	0.399	-0.095; 0.238	0.008	0.086	0.929	-0.161; 0.177
Education: University	0.110	0.068	0.108	-0.024; 0.244	0.054	0.069	0.432	-0.082; 0.190
bpConcreteness	0.081	0.012	<.0001	0.057; 0.105	0.075	0.013	<.0001	0.050; 0.099
bpPast Orientation	-0.036	0.012	0.003	-0.060; -0.012	-0.033	0.012	0.000	-0.057; -0.008

bpObsolescence	-0.033	0.014	0.020	-0.060; -0.005	-0.039	0.014	0.007	-0.067; -0.011
bpFinitude	0.007	0.010	0.481	-0.012;0.026	0.010	0.010	0.304	-0.009; 0.030
bpConcreteness*Age	0.001	0.010	0.271	-0.001; 0.003				
bpPast Orientation*Age	0.002	0.001	0.028	0.000; 0.004				
bpObsolescence*Age	0.001	0.001	0.408	-0.001; 0.003				
bpFinitude*Age	0.000	0.001	0.456	-0.001; 0.002				
bpConcreteness*SRLE					-0.0003	0.001	0.754	-0.002; 0.002
bpPast Orientation*SRLE					-0.001	0.001	0.274	-0.003; 0.001
bpObsolescence*SRLE					-0.001	0.001	0.433	-0.002; 0.001
bpFinitude*SRLE					-0.000	0.001	0.991	-0.001; 0.001
Time	-0.007	0.004	0.094	-0.016;0.001	-0.008	0.004	0.091	-0.016; 0.001
wpConcreteness	0.047	0.012	<.0001	0.025; 0.070	0.048	0.012	<.0001	0.025; 0.071
wpPast Orientation	0.008	0.011	0.440	-0.013: 0.030	0.008	0.011	0.496	-0.014; 0.029
wpObsolescence	-0.007	0.012	0.569	-0.031; 0.017	-0.006	0.012	0.612	-0.030; 0.018
wpFinitude	-0.011	0.011	0.308	-0.034; 0.011	-0.011	0.011	0.313	-0.034; 0.011
wpConcreteness*Age	-0.002	0.001	0.009	-0.004; -0.001				
wpPast Orientation*Age	-0.001	0.001	0.402	-0.002; 0.001				

wpObsolescence*Age	0.001	0.001	0.405	-0.001; 0.002				
wpFinitude*Age	0.001	0.001	0.167	-0.000; 0.003				
wpConcreteness*SRLE					0.001	0.001	0.092	-0.000; 0.003
wpPast Orientation*SRLE					0.000	0.001	0.579	-0.001; 0.002
wpObsolescence*SRLE					-0.000	0.001	0.650	-0.002; 0.001
wpFinitude*SRLE					-0.000	0.001	0.713	-0.002; 0.001
Random effects								
Variance intercept	0.057	0.023	.0007		0.076	0.042	.0008	
Variance slope	0.001	0.001	.117		0.001	0.001	0.063	
Cov. intercept, slope	0.003	0.003	.367		0.001	0.003	0.687	
Variance explained								
	.13				.14			

Note. *N* = 420 who provided 1,239 observations. bp = between–person. wp = within-person. CI = confidence intervals. Gender was coded as 0 = male, 1 = female. Self-rated health: Higher scores indicate better self-rated health. Education: reference group is vocational training. Income was rescaled in thousands so that a difference in income by one unit corresponds to a difference of 1000 Euro. Cov. = covariance. Unstandardized estimates and standard errors are presented. Chronological age was grand–mean–centered at 54.2 years, and subjective remaining life expectancy was grand-mean centered at 26.6 years.
