# Supplementary material for: Longitudinal associations between time perspective and life satisfaction across adulthood
Source: Commun Psychol. 2024 Jul 20;2:67. doi: 10.1038/s44271-024-00118-0 (PMC11332047; doi:10.1038/s44271-024-00118-0)
Supplement: Supplementary file 3 — Reporting Summary [file 44271_2024_118_MOESM3_ESM.pdf]

nature portfolio

Corresponding author(s): Maria Wirth, maria.wirth@uni-jena.de

Last updated by author(s): Jul 1, 2024

Reporting Summary

Nature Portfolio wishes to improve the reproducibility of the work that we publish. This form provides structure for consistency and transparency in reporting. For further information on Nature Portfolio policies, see our Editorial Policies and the Editorial Policy Checklist.

Statistics

For all statistical analyses, confirm that the following items are present in the figure legend, table legend, main text, or Methods section.

n/a	Confirmed	
☐	☒	The exact sample size (*n*) for each experimental group/condition, given as a discrete number and unit of measurement
☐	☒	A statement on whether measurements were taken from distinct samples or whether the same sample was measured repeatedly
☐	☒	The statistical test(s) used AND whether they are one- or two-sided<br>*Only common tests should be described solely by name; describe more complex techniques in the Methods section.*
☐	☒	A description of all covariates tested
☐	☒	A description of any assumptions or corrections, such as tests of normality and adjustment for multiple comparisons
☐	☒	A full description of the statistical parameters including central tendency (e.g. means) or other basic estimates (e.g. regression coefficient) AND variation (e.g. standard deviation) or associated estimates of uncertainty (e.g. confidence intervals)
☐	☒	For null hypothesis testing, the test statistic (e.g. *F*, *t*, *r*) with confidence intervals, effect sizes, degrees of freedom and *P* value noted<br>*Give P values as exact values whenever suitable.*
☒	☐	For Bayesian analysis, information on the choice of priors and Markov chain Monte Carlo settings
☐	☒	For hierarchical and complex designs, identification of the appropriate level for tests and full reporting of outcomes
☐	☒	Estimates of effect sizes (e.g. Cohen's *d*, Pearson's *r*), indicating how they were calculated

Our web collection on statistics for biologists contains articles on many of the points above.

Software and code

Policy information about availability of computer code

Data collection	no software was used for data collection, paper-pen only
Data analysis	SAS 15.3

For manuscripts utilizing custom algorithms or software that are central to the research but not yet described in published literature, software must be made available to editors and reviewers. We strongly encourage code deposition in a community repository (e.g. GitHub). See the Nature Portfolio guidelines for submitting code & software for further information.

Data

Policy information about availability of data

All manuscripts must include a data availability statement. This statement should provide the following information, where applicable:

- Accession codes, unique identifiers, or web links for publicly available datasets
- A description of any restrictions on data availability
- For clinical datasets or third party data, please ensure that the statement adheres to our policy

The raw data supporting the conclusions of this article will be made available by the authors upon request.

Human research participants

Policy information about studies involving human research participants and Sex and Gender in Research.

Reporting on sex and gender	The original sample was stratified by age cohort (1929–1938 × 1939–1948 × 1949–1958 × 1959–1968 × 1969–1978) and gender (male × female). Gender was based on self-reporting. The sample selected for analyses comprised 234 females and 225 males. No gender based analyses were performed.
Population characteristics	We controlled in our analysis for gender, primary education, income, and subjective health reported at T1. Subjective health was assessed by a single item, "How would you rate your current health?" with a response scale from 0 (very poor) to 4 (very good). For the analyses, primary education was dummy-coded, using Volks-/Hauptschulabschluss (< 10 years of formal education) as reference category. For those individuals who reported "other educational degree" (N = 6), the education score was set to missing. Two individuals reported an income that was more than 3 standard deviations above the sample mean. We considered their income scores as outliers and set them to missing.
Recruitment	Participants were recruited from two German cities, Jena and Erlangen, using information obtained from local registry offices.
Ethics oversight	Research procedures were approved by the Institutional Review Board at Friedrich-Schiller-University Jena (FSV 18/36)

Note that full information on the approval of the study protocol must also be provided in the manuscript.

Field-specific reporting

Please select the one below that is the best fit for your research. If you are not sure, read the appropriate sections before making your selection.

☐ Life sciences ☒ Behavioural & social sciences ☐ Ecological, evolutionary & environmental sciences

For a reference copy of the document with all sections, see nature.com/documents/nr-reporting-summary-flat.pdf

Behavioural & social sciences study design

All studies must disclose on these points even when the disclosure is negative.

Study description	Longitudinal, quantitative questionnaire study
Research sample	Individuals starting at age 30 living in Jena or Nürnberg, The sample included 459 participants aged 30–80 years at T1 (Mage = 54.22, SD = 13.80, 51% female). The study used stratified random sampling (balanced design) and the sample was stratified by age cohort (1929–1938 × 1939–1948 × 1949–1958 × 1959–1968 × 1969–1978) and gender (male × female). This is a secondary analysis of data collected within the aging as future project (AAF).
Sampling strategy	Participants were recruited from two German cities, Jena and Erlangen, using information obtained from local registry offices. This is a secondary analysis of existing data, hence we did not conduct a power analysis.
Data collection	Participants received the questionnaire via mail addressed to their homes. Individuals were told to complete the questionnaire on their own using a pen.
Timing	Data was collected in 2009, 2014, 2019
Data exclusions	For those individuals who reported "other educational degree" (N = 6), the education score was set to missing. Two individuals reported an income that was more than 3 standard deviations above the sample mean. We considered their income scores as outliers and set them to missing.
Non-participation	The initial sample at T1 comprised N = 768; 120 individuals only participated in T1, 71 in T2, and 112 only in T3. 134 individuals participated only in T1 and T2, 55 in T1 and T3, and 164 in T2 and T3. We observed no systematic differences between AAF participants who participated only in T1 compared to those who took part in more than one measurement occasion (Park et al., 2022).
Randomization	no randomization

Reporting for specific materials, systems and methods

We require information from authors about some types of materials, experimental systems and methods used in many studies. Here, indicate whether each material, system or method listed is relevant to your study. If you are not sure if a list item applies to your research, read the appropriate section before selecting a response.

Materials & experimental systems

n/a	Involved in the study
☒	☐ Antibodies
☒	☐ Eukaryotic cell lines
☒	☐ Palaeontology and archaeology
☒	☐ Animals and other organisms
☒	☐ Clinical data
☒	☐ Dual use research of concern

Methods

n/a	Involved in the study
☒	☐ ChIP-seq
☒	☐ Flow cytometry
☒	☐ MRI-based neuroimaging
